# Prevalence of exclusive breastfeeding practice and its association with maternal employment in Ethiopia: a systematic review and meta-analysis

**DOI:** 10.1186/s13006-021-00432-x

**Published:** 2021-10-30

**Authors:** Getu Engida Wake, Yohannes Moges Mittiku

**Affiliations:** College of Health Science, Debre Berhan University, Debre Berhan, Ethiopia

**Keywords:** Prevalence, Exclusive breastfeeding, Meta-analysis, Maternal employment, Ethiopia

## Abstract

**Background:**

Exclusive breastfeeding is defined as the practice of providing only breast milk for an infant for the first 6 months of life without the addition of any other food or water, except for vitamins, mineral supplements, and medicines. Findings are inconsistent regarding the prevalence of exclusive breastfeeding in Ethiopia. Full-time maternal employment is an important factor contributing to the low rates of practice of exclusive breastfeeding. Empowering women to exclusively breastfeed, by enacting 6 months’ mandatory paid maternity leave can increase the rate of exclusive breastfeeding in the first 6 months of life up to 50%. The purpose of this review was to estimate the pooled prevalence of exclusive breastfeeding and its association with full-time maternal employment in the first 6 months of life for infants in the context of Ethiopia.

**Methods:**

The Preferred Reporting Items for Systematic Reviews and Meta-Analyses (PRISMA) guideline was used in this systematic review and meta-analysis. All observational studies reporting the prevalence of exclusive breastfeeding and its association with maternal employment in Ethiopia were considered. The search was conducted from 6 November 2020 to 31 December 2020 and all papers published in the English language from 1 January 2015 to 31 December 2020 were included in this review.

**Results:**

Forty-five studies were included in the final analysis after reviewing 751 studies in this meta-analysis yielding the pooled prevalence of EBF 60.42% (95% CI 55.81, 65.02) at 6 months in Ethiopia. Those full-time employed mothers in the first 6 months were 57% less likely to practice exclusive breastfeeding in comparison to mothers not in paid employment in Ethiopia (OR 0.43; 95% CI 0.31, 0.61).

**Conclusions:**

Full-time maternal employment was negatively associated with the practice of exclusive breastfeeding in comparison to unemployed mothers. The prevalence of exclusive breastfeeding in Ethiopia is low in comparison to the global recommendation. The Ethiopian government should implement policies that empower women. The governmental and non-governmental organizations should create a conducive environment for mothers to practice exclusive breastfeeding in the workplace.

**Supplementary Information:**

The online version contains supplementary material available at 10.1186/s13006-021-00432-x.

## Background

Breastfeeding is a core part of the 2030 agenda for Sustainable Development Goals (SDG) which is linked with many targets of the SDGs, especially with the third target which deals with ending preventable maternal and neonatal death [1]. Exclusive breastfeeding (EBF) is defined as the practice of providing only breast milk for an infant for the first 6 months of life without the addition of any other food or water, except for vitamins, mineral supplements, and medicines [2]. Currently, the global prevalence of EBF for infants aged zero to 6 months is only 38%, which is far behind to making EBF during the first 6 months of life the norm for infant feeding. Researchers indicate that 11.6% of mortality in children under 5 years of age was contributed by non-exclusive breastfeeding [3, 4].

In 2012, the World Health Assembly endorsed a comprehensive implementation plan on maternal, infant, and young child nutrition with six specified global nutrition targets for 2025.The fifth target aims to increase the rate of EBF in the first 6 months up to 50% and only 31 of 194 countries were practicing according to this endorsement in 2018 [5, 6]. According to the 2015 UNICEF report, the worldwide rate of EBF is low compared to the 2012 World Health Assembly endorsement, with the following EBF rates reported in western and central Africa (25%), East Asia and Pacific (30%), South Asia (47%), Central America and the Caribbean (32%), eastern and southern Asia (51%), least developed countries (46%) and worldwide (38%) respectively [7]. Between 1985 and 1995, global rates of EBF were raised by 2.4%, however, 25 countries raised their rates of EBF by 20% or more after 1995 [8, 9]. Similarly, Cambodia and Malawi showed an increment of EBF from 11 to 74% and 3 to 71% respectively between 1992 and 2010 [10].

Another study conducted in 13 western African countries and sub-Saharan countries showed the prevalence of EBF for infants under 6 months of age ranges from 13.0% in Côte d’Ivoire to 58.0% in Togo and 45.2% in sub-Saharan countries respectively [11, 12]. Besides this, according to the 2016 Ethiopian demographic health survey (EDHS), the prevalence of EBF for infants under 6 months was 58% [13]. According to a study conducted in Latin America and the Caribbean countries, Bangladesh and others, EBF for the first 3 months of life can prevent 55% of infant deaths related to diarrheal disease and acute respiratory infection [14–17].

Similarly, a study conducted in Ghana and Ethiopia showed that the risk of neonatal death was higher for infants with non-exclusive breastfeeding [18, 19]. Inadequate rates of EBF result from different factors such as inadequate maternity leave (shorter paid maternity leave which enforces mother to return to work early before 6 months of infant’s age) [20, 21], workplace policies that don’t support a woman’s ability to breastfeed when she returns to work [22], and caregiver and societal belief which favor non-exclusive breastfeeding before 6 months of age of infants [23–25]. Some evidence showed that empowering women to exclusively breastfeed, by enacting 6 months’ mandatory paid maternity leave, as well as policies that encourage women to breastfeed in the workplace and public can increase the rate of EBF in the first 6 months of life up to 50% [26, 27]. Another piece of evidence showed that longer paid maternity leave helps the mothers to practice EBF effectively [28].

The Indian and Vietnamese governments have been successfully protecting EBF by the implementation of supportive policies that guarantee mothers get 6 months of paid maternity leave and by prohibiting the use of marketing breast milk substitutes with legislation before 6 months of infant’s age [29, 30]. Whereas, contrary to the World Health Organization’s recommendation, the Constitution of Ethiopia and Labour Proclamation recommends employed mothers get fully paid maternity leave of 120 working days only (30 days antenatal and 90 days postnatal leave) and the proclamation doesn’t support women to breastfeed in the workplace and the public area after they return to work [31].

In Ethiopia, many studies have been conducted to determine the prevalence of EBF and its associated factors between 1 January 2015 to 31 December 2020. These studies showed that different maternal and health service-related factors influenced the practice of EBF in addition to maternal employment [32–50]. We selected maternal employment from other factors to investigate its effect on the practice of EBF because of the following reasons: The first reason is that maternal employment was an important factor, which ultimately influences EBF, especially in our country where the Labour Proclamation recommends only 120 working days paid maternity leave which forces mothers to return quickly to their job before 6 months after delivery. The second reason is that the primary studies conducted previously found inconsistent evidence regarding the effect of maternal employment on EBF. Most showed a negative association of maternal employment with EBF with the presence of great variation among them [32–37, 40–50]. Only two studies [38, 39] showed a positive association of maternal employment with EBF.

As far as we are aware, even if there were small and fragmented studies, there is no published systematic review and meta-analysis in Ethiopia, which has investigated the pooled prevalence of EBF and its association with maternal employment using primary studies published between 1 January 2015 to 31 December 2020, which is in line with the third target of the SDGs by 2030. The objective of this systematic review and meta-analysis was to estimate the pooled prevalence of EBF and its association with full-time maternal employment in the context of Ethiopia. This systematic review will generate concrete evidence that helps policymakers and program planners to make an appropriate intervention and remold some policies concerning maternal employment and the practice of EBF for the benefit of mothers and infants in Ethiopia.

## Methods

The current systematic review and meta-analysis was reported by using the Preferred Reporting Items for Systematic Reviews and Meta-Analysis (PRISMA) [51] guideline to determine the pooled prevalence of EBF practice and its association with maternal employment.

### Research question / hypothesis according to CoCoPop (condition, context, population) criteria

What is the prevalence of Exclusive breastfeeding (EBF) and its association with full-time maternal employment among mothers with infants less than 5 years of age in the context of Ethiopia?

### Searching strategies

The international databases, including PubMed, Google Scholar, Science Direct, and Cochrane library, Scopus, CINAHL, and Web of Science were systematically searched. The search was conducted using the following keywords: “Prevalence”, “Exclusive Breastfeeding”, “Feeding, Breast”, “Breastfeeding”, “Breastfeeding, exclusive”, “Factors”, “Determinants”, “Maternal employment”, and Ethiopia. The search terms were used separately and in combination using Boolean operators including “OR” or “AND” and the search was conducted from 6 November 2020 to 31 December 2020. All papers published until 31 December 2020 were included in this review.

### Eligibility criteria

#### Inclusion criteria

Study area: Only studies conducted in Ethiopia.

Publication condition: Articles published in peer-reviewed journals.

Study design: All observational study designs (Cross-sectional, case-control, and cohort) reporting the prevalence of EBF or associations between maternal employments with EBF were considered.

The outcome of interests: Studies reported data on the prevalence of EBF or the association between EBF and maternal employment were considered.

Language: Articles reported in the English language were considered.

Publication year: only studies published from 1 January 2015 to 31 December 2020 were considered.

#### Exclusion criteria

- Study conducted in women with HIV / AIDS, preterm newborn, and newborn in an intensive care unit
- Study with abstracts without full text and qualitative studies, symposium / conference and case reports.
- Articles, which were not fully accessed, after at least two email contacts with the primary author, were excluded and experimental, intervention, and review articles were excluded.

### Outcome measurement

This systematic review has two main outcomes. The first one is the prevalence of EBF practice, which is defined as the practice of providing only breast milk for an infant for the first 6 months of life without the addition of any other food or water, except for vitamins, mineral supplements, and medicines [2]. The prevalence was calculated from each primary study by dividing the number of women breastfeeding exclusively by the total number of all women who had participated in the study multiplied by 100. The second outcome was to investigate the association between full-time maternal employment and the practice of EBF for which we calculated the Ln odds ratio (Ln OR) meaning the base e logarithm or (loge (OR)) results of the primary studies that examined the relationship between maternal employment and practice EBF.

#### Data extraction

Two authors (GE and YM) independently assessed the quality of each original study and any disagreements at the time of data abstraction were resolved through discussion and consensus. Data were extracted using a standardized data extraction format, which was adopted from the Joanna Briggs Institute (JBI) data extraction format [52]. The following data such as primary authors, publication year, and study area, study design, sample size, the prevalence of exclusive breastfeeding, the quality score of each study, the association between maternal employment and EBF with their respective odds ratio (OR), characteristics of study participants and response rate were extracted.

#### Quality assessment

The Joanna Briggs Institute Critical Appraisal tools for use in JBI Systematic Reviews (JBI-MAStARI) was used for critical appraisal of studies [53]. The tool has eight major criteria for critical appraisal of each primary study. Accordingly, primary studies with a score of equal or greater than 50% and above were included in the meta-analysis research.

#### Statistical analysis

Data were extracted in Microsoft Excel format and analysis was done using STATA version 11 software. We calculated the standard error for each original study using the binomial distribution format. Heterogeneity regarding reported prevalence was assessed by computing *p*-values for Cochrane Q-statistics and I^2^ tests. I^2^ test statistics of 25, 50, and 75% were declared as low, moderate, and high heterogeneity respectively [54]. The test statistic showed that there was significant heterogeneity among the included studies (98.8%; p = < 0.001), and because of this a random-effects meta-analysis model was used to estimate the DerSimonian and Laird pooled effect. To minimize the random variations between primary studies, subgroup analysis was done by region in Ethiopia, sample size, and publication year of primary studies. Besides the above, univariate meta-regression was conducted by considering the same three subgroups as covariates to identify the possible sources of heterogeneity, but none was found to be statistically significant.

We checked publication bias by funnel plot subjectively and Egger’s and Begg’s tests objectively; a *p*-value of less than 0.05 was used to declare the statistical significance of publication bias [55]. For this meta-analysis pooled prevalence of EBF with a 95% confidence interval (CI) was presented with the forest plot. Accordingly, the size of each box corresponds to the weight of the study, the crossed line refers to a 95% confidence interval of the study, and the Ln OR which is the base e logarithm was applied to examine the association between maternal employment and EBF in Ethiopia.

## Results

**Fig. 1:**
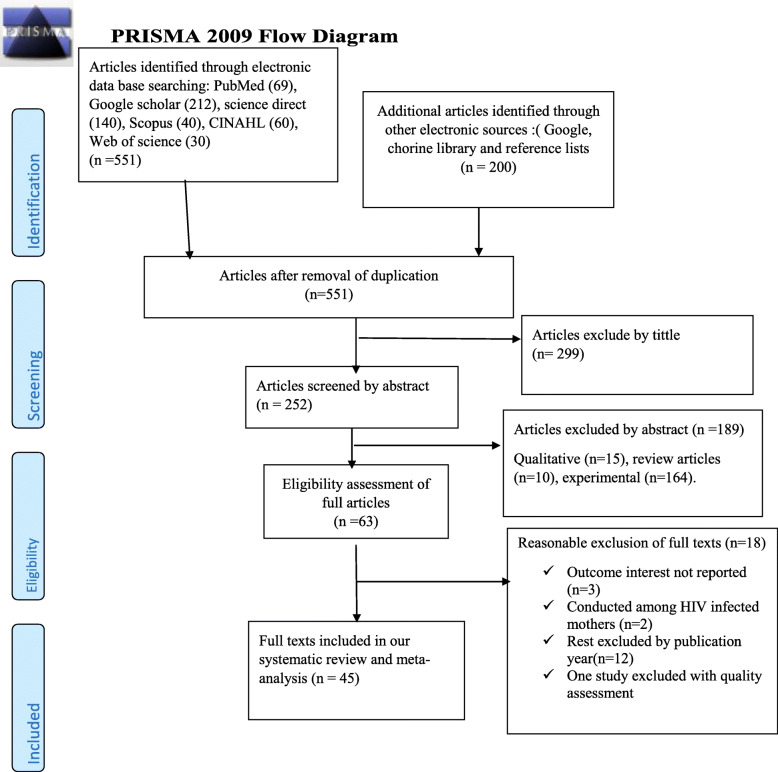
Flow diagram of studies included in systematic review and meta-analysis, 2020

As described in Fig. 1, 751 studies were identified regarding EBF in Ethiopia through PubMed, Google Scholar, Science Direct, Scopus, CINAHL, Web of Science, and others in the first step. Then 200 studies were excluded because of duplication. From the remaining 551 studies, a further 299 articles were excluded as being not relevant to this review on the basis of their titles. The remaining 252 studies were screened by their abstracts yielding an additional 189 studies to be excluded. Moreover, 63 full-text articles were assessed for eligibility based on the preset inclusion criteria, and from these, 18 articles were excluded due to the inclusion criteria. Finally, 45 studies fulfilled the inclusion criteria and were included in the systematic review and meta-analysis.

As shown in Table 1, all 45 of these studies were published between 1 January 2015 to 31 December 2020. Regarding study design most 42 of the studies are cross-sectional study designs. The sample size of the studies ranges from 226 to 5227. The lowest prevalence (29.29%) of EBF was observed in a study conducted in Addis Ababa, Ethiopia [72] whereas the highest prevalence (87.84%) was observed in a study conducted in the Southern Nations, Nationalities, and Peoples (SNNP) and Tigray region, among rural mothers in Ethiopia [81]. From nine regions of Ethiopia, seven regions and two council cities were represented in this meta-analysis. Fifteen of the studies were from Amhara [33–42, 58, 59, 75–77] two from Addis Ababa [64, 73], four from Affar [34, 56, 57, 74] four from Oromia [44, 60, 61, 80], twelve from SNNP [45–47, 62–65, 67–70, 72], one from Tigray [71], two from Somalia [48, 79], two from Harari [43, 78], one from nationwide [49], one from Diredawa [50] and one from SNNP and Tigray [81]. No studies were reported from Benishangul Gumiz and Gagmbela in this review research. Regarding the quality score of each primary study, the score was between the lowest (4) and highest (8) (Additional file 1) and almost all primary studies had a sufficient response rate.

**Table 1:** Descriptive summary of 45 studies included in the meta-analysis for estimation of pooled prevalence of exclusive breastfeeding in Ethiopia, 2020

s.no	Primary author	Publication year	Study area	Study design	Sample size	Prevalence of EBF (%)	Response rate (%)	Association of maternal employment with EBF (OR)	Study participants characteristics
1	Tsegaye et al. [32]	2019	Affar	cross-sectional	618	55	98	0.8	mothers who had a child age < 6 months
2	Liben et al. [56]	2016	Affar	cross-sectional	333	81	96.2	–	Mothers who had child age < 6 months
3	Gizaw et a [57]	2017	Affar	cross-sectional	254	74	98.5	–	Mothers who had child age between 6 and 24 months during the first 6 months of life
4	Asemahagn [33]	2016	Amhara	cross-sectional	332	78.9	96	0.6	Mothers who had child age 0-6 months
5	Belachew et al. [34]	2018	Amhara	cross-sectional	472	86.44	94.6	0.334	Mothers who had child age < 6 months
6	Biks et al. [58]	2015	Amhara	Case-control	1769	30.69	NR	–	mothers who exclusively breastfed their infants for the first six months were selected As cases.
7	Tariku et al. [59]	2017	Amhara	Demographic Surveillance	5227	54.5	NR	–	mothers with children aged less than 59 months
8	Asfaw et al. [35]	2015	Amhara	cross-sectional	634	68.6	100	0.36	Mothers who had child age < 12 months
9	Yeshamble Sinshaw et al. [36]	2015	Amhara	cross-sectional	483	61.28	100	0.4	Mothers who had child age < 6 months
10	Mekuria et al. [37]	2015	Amhara	cross-sectional	413	60.77	97.6	0.5	Mothers who had child age < 6 months
11	Arage et al. [38]	2016	Amhara	cross-sectional	453	70.86	96.4	1.07	Mothers who had child age < 6 months
12	Gebrie et al. [39]	2019	Amhara	cross-sectional	254	46.45	NR	2.452	mothers who had a child up to one year
13	Chekol et al. [40]	2017	Amhara	cross-sectional	649	34.82	100	0.29	mothers who had a child age 7–12 months
14	Hunegnaw et al. [41]	2017	Amhara	cross-sectional	478	74.26	94.4	0.49	mothers who had a child age 6–12 months
15	Tewabe et al. [42]	2017	Amhara	cross-sectional	405	50.12	95.7	0.33	Mother who had child age < 6 months
16	Iffa et al. [43]	2018	Harar	cross-sectional	425	40.94	100	0.1	mothers who had a child age 0–31 months
17	Bayissa Z B. et al. [44]	2015	Oromia	cross-sectional	371	82.21	92.05	0.41	mothers who had child age < 2 years
18	Kitesa et al. [60]	2017	Oromia	cross-sectional	2222	44.32	100	–	mothers who had a child age ≤ 12 months
19	Sasie D et al. [61]	2017	Oromia	cross-sectional	410	70	97.4	–	mothers who had a child age 0–23 months
20	Anjullo B et al. [62]	2018	SNNP	cross-sectional	330	53.93	100	–	mothers who had child age < 6 months
21	Muze Edris MD, et al. [45]	2019	SNNP	cross-sectional	843	56.10	99.7	0.77	mothers who had child age < 23 months
22	Gedion Asnake Azeze et al. [63]	2019	SNNP	cross-sectional	403	64.76	97.8	–	mothers who had child age 6-12 months
23	Sorato M [64]	2017	SNNP	cross-sectional	226	40.70	92	–	mothers who had child age 0–12 months
24	Reddy S et al. [65]	2016	SNNP	cross-sectional	347	57.63	98.02	–	mothers who had child age under2years
25	Bisrat et al. [66]	2017	SNNP	cross-sectional	765	49.15	90.6	–	mothers who had child age < 6 months
26	Sonko A et al. [67]	2015	SNNP	cross-sectional	420	70.47	99.5	–	mothers who had child age < 6 months
27	Adugna et al. [46]	2017	SNNP	cross-sectional	529	60.86	97.8	0.4	mothers who had child age0- 6 months
28	Alemu Earsido.et al. [68]	2017	SNNP	cross-sectional	707	73.83	98	–	mothers who had child age 0–12 months
29	Eskezyiaw Agedew Getahu.et al. [47]	2017	SNNP	cross-sectional	562	40.56	99.11	0.44	mothers who had child age 6-24 months
30	Lenja et al. [69]	2016	SNNP	cross-sectional	396	78.03	98	–	mothers who had child age < 6 months
31	Kelaye T [70]	2017	SNNP	cross-sectional	421	64.84	100	–	mothers who had child age < 6 months
32	Tadesse et al. [48]	2019	Somalia	cross-sectional	558	71.14	95.7	0.04	mothers who had child age 3-5 months
33	Teka et al. [71]	2015	Tigray	cross-sectional	530	70.18	98	–	mothers who had child age < 24 months
34	Shifraw et al. [72]	2015	Addis Ababa	cross-sectional	635	29.29	98	–	mothers who had child age ≤ 9 months
35	Elyas l [73]	2017	Addis Ababa	cross-sectional	380	44.21	90.3	–	mothers who were breastfeeding and visited the clinic pediatric clinic
36	Ahmed et al. [49]	2019	EDHS based data	(EDHS) base data	3861	59.90	NR	0.94	mothers who had child age 0–23 months
37	Nur et al. [74]	2018	Affar	cross-sectional	400	78.3	97.3	–	Mothers who had infants aged 0–6 months
38	Tilksew Ayalew [75]	2020	Amhara	cross-sectional	400	57.3	95	–	Mothers who had infants aged 0–6 months
39	Alebachew et al. [76]	2017	Amhara	cross-sectional	332	49.7	100	–	Mothers who had infants aged less than 2 years
40	Desalew et al. [50]	2020	Diredawa	cross-sectional	704	81.1	100	0.52 (0.321,0.85)	Mothers who had infants aged 6–23 months
41	Bazie et al. [77]	2019	Amhara	cross-sectional	608	46.7	95.9	–	Mothers who had infants aged 6–12 months
42	Dibisa et al. [78]	2020	Harar	cross-sectional	577	45.8	97.8	–	Mothers who had infants aged less than 12 months
43	Musse Obsiye [79]	2019	Somalia	cross-sectional	570	54.91	96.28	–	Mothers of Infants Aged Under Six Months
44	Mamo et al. [80]	2020	Oromia	cross-sectional	710	65.4	97.9	–	Mothers who had infants aged 6–9 months
45	Hagos et al. [81]	2020	SNNP and Tigray	cross-sectional	584	88.00	97.33	–	Mothers of Infants Aged Under Six Months

### Meta-analysis

#### Pooled prevalence of exclusive breastfeeding in Ethiopia

**Fig. 2:**
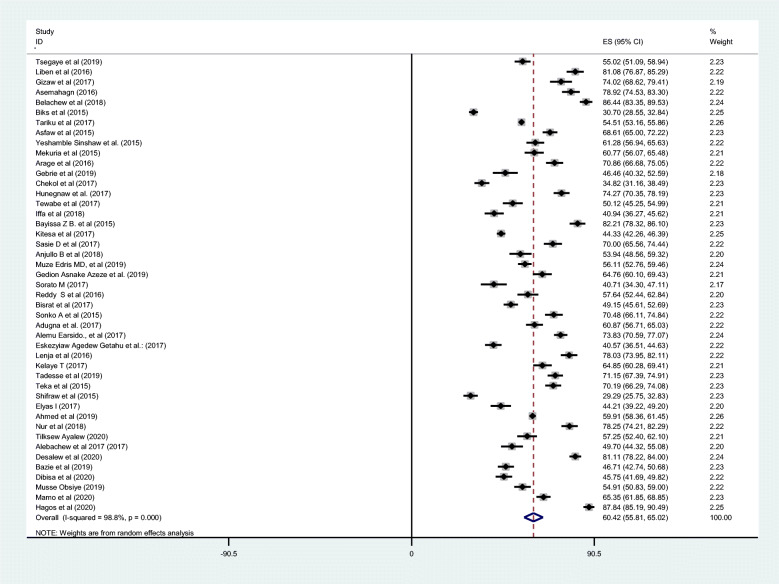
Forest plot displaying the pooled prevalence of exclusive breastfeeding of 45 studies in Ethiopia, 2020

**Fig. 3:**
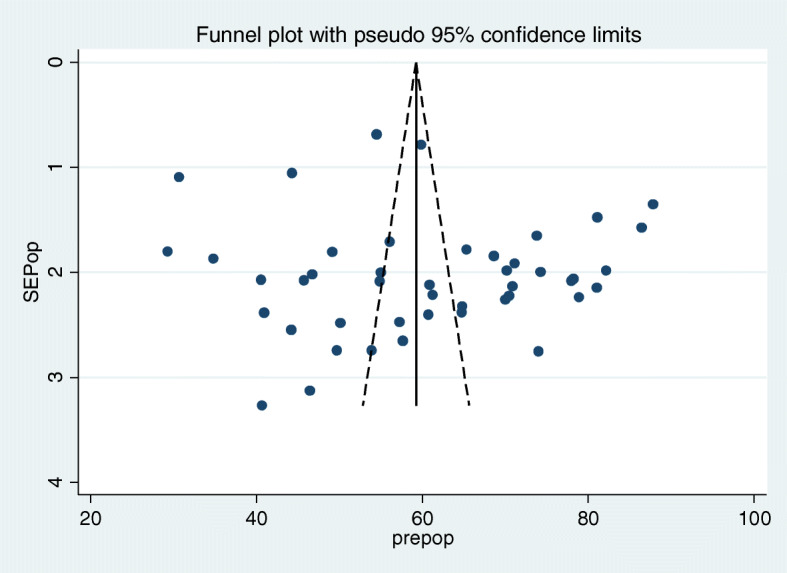
Funnel plot for publication bias, with PREPOP represented in the x-axis and standard error of SEPOP on the y-axis, 2020

A total of 45 studies of 33,000 breastfeeding women were included to estimate the pooled prevalence of EBF in the current meta-analysis. The pooled prevalence of EBF at 6 months was 60.42% (95% CI: 55.81, 65.02). The I2 test result indicated high heterogeneity among included studies (I2 98.8%; p = < 0.001), and because of this high heterogeneity the random effect meta-analysis model was used (Fig. 2). We also conducted a univariate meta-regression by considering the year of publication, sample size, and region in Ethiopia as covariates to identify the possible sources of heterogeneity, and unfortunately, none was found to be statistically significant (Table 2). Additionally, publication bias was assessed subjectively and objectively using both a funnel plot and Begg’s and Egger’s tests respectively. Even if the funnel plot showed the presence of publication bias (Fig. 3), no publication bias was found according to the results of Begg’s and Egger’s tests for the prevalence of EBF (*p* = 0.304) and (*p* = 0.314) respectively.

**Table 2:** heterogeneity of exclusive breastfeeding prevalence in the current meta-analysis (based on univariate meta-regression considering Year of publication, Sample size, and Regions in Ethiopia as a covariate), 2020

Variables	Coefficient (individual)	***p***-value (individual)
Year of publication	0.0159	0.896
Sample size	0.000229	0.323
Regions in Ethiopia	0.00339	0.960

#### Subgroup analysis

Subgroup analyses were conducted by splitting all primary studies included in the analysis by region (geographical locations) in Ethiopia, the total sample size, and publication year, to make comparisons between them and as a means of investigating heterogeneity. Accordingly, this systematic review and meta-analysis showed that the highest prevalence of EBF was reported in a study conducted in SNNP and Tigray 87.84% (95% CI: 85.19, 90.48), a study published during (2015–2016) 64.60% (95% CI: 52.90, 76.30) and among studies with a sample size with less than 500 participants (64.15% (95% CI: 58.61, 69.68)) respectively (Table 3).

**Table 3:** The subgroup analysis for the prevalence of exclusive breastfeeding by region and year of publication and sample size in Ethiopia, 2020 (*n* = 45)

Variables	Characteristics	Number of Included study	Prevalence (95% CI)	I2	***p***-value
**By region**	Addis Ababa	2	36.64 (22.02,51.26)	95.6%	< 0.001
	Affar	4	72.07 (59.61,84.53)	97.0%	< 0.001
	Amhara	15	58.10 (49.50,66.71)	99.0%	< 0.001
	Harari	2	43.49 (38.78,48.20)	56.9%	< 0.001
	Nationwide	1	59.91 (58.36,61.45)	–	–
	Oromia	4	65.43 (47.29,83.56)	99.2%	< 0.001
	SNNP	12	59.31 (52.49,66.14)	96.8%	< 0.001
	Somalia	2	63.05 (47.14,78.96)	97.0%	< 0.001
	Tigray	1	70.19 (66.29,74.08)	–	–
	Dire Dawa	1	81.11 (78.22,84.00)	–	–
	SNNP and Tigray	1	87.84 (85.19,90.48)	–	–
**By the year of publication**	2015–2016	13	64.60 (52.90,76.30)	99.2%	< 0.001
	2017–2019	32	58.74 (53.89,63.59)	98.5%	< 0.001
**By sample size**	< 500	24	64.15 (58.61,69.68)	97.2%	< 0.001
	500–1000	17	58.33 (49.99,66.67)	98.9%	< 0.001
	≥1000	4	47.38 (35.86,58.90)	99.4	< 0.001

### Association between maternal employment and exclusive breastfeeding in Ethiopia

**Fig. 4:**
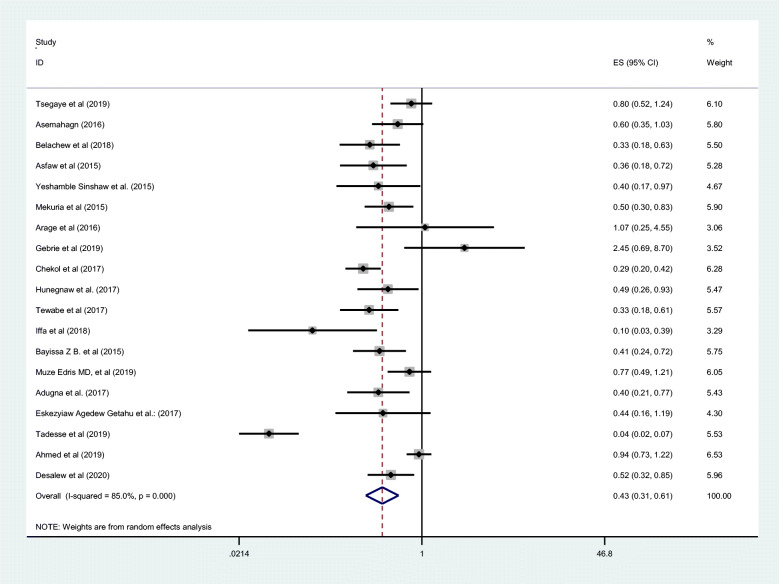
The pooled odds ratio of the association between maternal employment and exclusive breastfeeding in Ethiopia in 2020

**Fig. 5:**
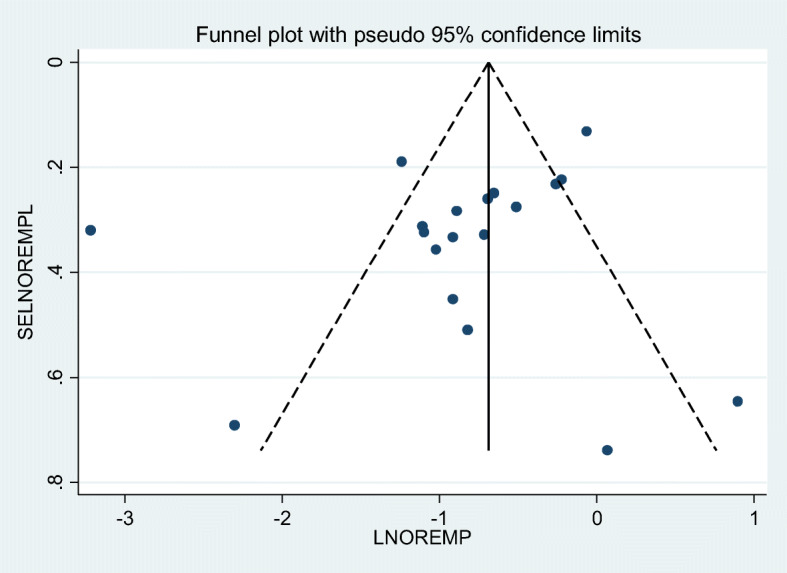
Funnel plot for publication bias, with LNOREMP represented in the x-axis and standard error of LNOREMP on the y-axis, 2020

We examined the association between maternal employment and EBF practice using 19 studies [32–50] in this meta-analysis and the findings showed that the practice of EBF was negatively associated with maternal employment (OR 0.43; 95% CI 0.31, 0.61). High heterogeneity (I2 = 85.0% and *p*-value < 0.000) was observed across the included studies and a random effect meta-analysis model was applied to examine the association between maternal employment and EBF in Ethiopia (Fig. 4). We also assessed publication bias subjectively using the funnel plot and objectively using Begg’s and Egger’s tests. While the funnel plot showed the presence of publication bias, Begg’s and Egger’s tests showed the absence of significant publication bias (*p*- value = 0.363 and *p* = 0.684) respectively (Fig. 5).

## Discussion

This systematic review and meta-analysis research was conducted to determine the pooled prevalence of EBF and its association with maternal employment in Ethiopia using a study published between 1 January 2015 and 31 December 2020. According to the results of 45 studies included in this meta-analysis, the pooled prevalence of EBF in Ethiopia is 60.42% (95% CI: 55.81, 65.02). The overall prevalence of EBF in this study is similar to the result of the 2016 Ethiopian Demographic and Health Survey (EDHS) result (58%) [13], and the result of a meta-analysis conducted in Ethiopia (59.3%) [82]. This finding could be attributed to similarities in socio-demographics, methodologies, and the characters of individual studies included in both review and EDHS reports. But, the overall reported prevalence of EBF in this review is higher than the result of meta-analysis result conducted in Iran (49.1%) [83] and 29 Sub-Saharan African (SSA) countries, which showed the prevalence of EBF to be 23.70% in Central Africa and 56.57% in Southern Africa [84]. The pooled prevalence of EBF in this review is also higher than the results of the study conducted in 27 Sub-Saharan African countries (36%) [85], the Demographic and Health Survey of Tanzania (22.9%) [86], Demographic and Health Survey of Madagascar (48.8%) [87] and the study conducted in developing countries (39%) [88]. This variation might be because of methodological differences, differences in infants and maternal socio-demographic characteristics, economics, health service utilization, the gap of the year in which the study was conducted, and the number of studies included in the review. But the overall prevalence of EBF in our review is lower than the result of the primary study conducted in Indian regions, which indicated the prevalence of EBF was 79.2% in southern India and 68.0% in northeastern India respectively [89], the Nepal Demographic and Health Survey result was 66.3% [90], and the result of the study conducted in Ghana, 64% [91].

Based on the subgroup analysis, the highest (87.84%) and lowest (36.64%) prevalence of EBF was reported in a study conducted among rural mothers of SNNP and Tigray region and Addis Ababa City respectively. This regional variation might be because of differences in socio-demographics, and the difference in numbers of the studies included in the two regions during analysis. In addition to the above, the participants of a study conducted in SNNP and Tigray region were rural resident mothers, and according to different kinds of literature being rural in residence for breastfeeding mothers is associated with a high prevalence of EBF practice [66, 77, 92].

We also performed a subgroup analysis using a year of study publication. Accordingly, the highest (64.60%) and lowest (58.74%) prevalence of EBF were reported in studies published during 2015–2016 and 2017–2020 respectively. This difference could be attributed to the difference in coverage of health information regarding EBF and effective utilization of health extension workers, adherence to the national and international policy by health institutions [93]. Besides, we conducted subgroup analysis using the total sample size of the study, and the highest (64.15%) and lowest (47.38%) prevalence of EBF was reported among studies with a sample size less than 500 and greater than or equal to 1000 respectively. This difference might be associated with a difference in the number of primary studies included in each category during analysis (24 primary studies in the category of the sample size of < 500 and four primary studies in the category of the sample size of ≥1000) respectively. One of the greatest threats to the validity of meta-analytic results is publication bias which generally leads to effect sizes being overestimated and the dissemination of false-positive results [94–96] and because of this, we assessed publication bias and possible sources of heterogeneity using Begg’s and Egger’s tests and univariate meta-regression respectively and no publication bias was found.

Full-time maternal employment was negatively associated with the practice of EBF among mothers who returned to work before 6 months in this systematic review and meta-analysis research (OR 0.43; 95% CI 0.31, 0.61). This result is in line with the results of a study conducted in 19 developing countries [97], another study conducted in Iran [98], a study conducted in developing countries [99], and a final study conducted in low and middle-income countries [100]. This similarity could be attributed to mothers who returned to work before 6 months postnatally and who have less frequent contact with their baby and employed mothers who begin liquid and solid based supplementation of food before the recommended age of starting weaning food which will result in the decreased practice of EBF [101]. Some evidence showed that employed mothers face unique barriers to practice EBF and returning to work too early after birth has been shown to affect the practice of EBF. Different kinds of literature showed that the more we increase the legislated duration of paid maternity leave, the more the mothers’ practice EBF and this will result in the higher prevalence of EBF [20, 102, 103].

### Limitations of the study

This review has certain limitations. The majority of the primary studies included in the review were cross-sectional studies which might affect the outcome variable because of other confounding factors. Studies published in a language other than English were not included in the review and the review addressed only one associated factor (maternal employment) with EBF. The review included some studies with a small sample size which might affect the pooled report of EBF. The last the last limitation is that the study protocol was not registered at the international prospective register of systematic reviews (PROSPERO).

## Conclusions

Full-time maternal employment was negatively associated with the practice of EBF in comparison to unemployed mothers. The prevalence of EBF in Ethiopia is low in comparison to the global recommendation. Based on our review findings, we recommended that the Ethiopian government should increase legislated paid maternity leave after delivery beyond currently paid maternity leave and implement policies that empower women. The governmental and non-governmental organizations should create a conducive environment for employed mothers to practice EBF at the workplace.

## Supplementary Information

**Additional file 1.** Quality score of included and excluded studies in this review to estimate the pooled prevalence of exclusive breastfeeding in Ethiopia, 2020.

## Data Availability

Datasets used for this study and other supplementing materials are available from the corresponding author on request.

## Abbreviations

AIDS: Acquired Immune Deficiency Syndrome
CI: Confidence Interval
EBF: Exclusive breastfeeding
EDHS: Ethiopian demographic health survey
HIV: Human immunodeficiency virus
HSTP: Health Sector Transformation Plan
JBI: Joanna Briggs Institute
JBI-MAStARI: Joanna Briggs Institute Meta-Analysis of Statistics Assessment and Review Instrument
MMR: Maternal mortality ratio
NM: Neonatal mortality
OR: Odds ratio
PRISMA: Preferred Reporting Items for Systematic Reviews and Meta-Analyses
PROSPERO: International Prospective Register of Systematic Reviews
SDG: Sustainable development goal
SNNP: Southern Nations, Nationalities, and Peoples
SSA: Sub-Saharan Africa
UN: United Nations
UNICEF: United Nations International Children’s Emergency Fund
WHO: World Health Organization
