# Supplementary material for: Prevalence of exclusive breastfeeding practice and its association with maternal employment in Ethiopia: a systematic review and meta-analysis
Source: Int Breastfeed J. 2021 Oct 30;16:86. doi: 10.1186/s13006-021-00432-x (PMC8557507; doi:10.1186/s13006-021-00432-x)
Supplement: Supplementary file 1 — Additional file 1. Quality score of included and excluded studies in this review to estimate the pooled prevalence of exclusive breastfeeding in Ethiopia, 2020. [file 13006_2021_432_MOESM1_ESM.docx]

Additional file 1: Table of quality score of included and excluded studies in this review to estimate the pooled prevalence of exclusive breastfeeding in Ethiopia, 2020.

|  |  | Assessment criteria's for quality of the primary studies | | | | | | | | |  |  |
| --- | --- | --- | --- | --- | --- | --- | --- | --- | --- | --- | --- | --- |
| s/no | studies | (1): Were the criteria for inclusion in the sample clearly defined?  1=Yes  0= No | (2): Were the study subjects and the setting described in detail?  1=Yes  0= No | (3): Was the exposure measured in a valid and reliable way?  1=Yes  0= No | (4): Were objective, standard criteria used for measurement of the condition?  1=Yes  0= No | (5): Were confounding factors identified?  1=Yes  0= No | (6): Were strategies to deal with confounding factors stated?  1=Yes  0= No | (7): Were the outcomes measured in a valid and reliable way?  1=Yes  0= No | (8): Was an appropriate statistical analysis used?  1=Yes  0= No | Total grade in number(summation of 1-8) | 100% | Rank |
| 1 | Tsegaye et al[32] | 1 | 1 | 1 | 1 | 0 | 1 | 1 | 1 | 7 | 87.5 | high |
| 2 | Liben et al[33] | 1 | 1 | 0 | 0 | 1 | 1 | 1 | 1 | 6 | 75 | medium |
| 3 | Gizaw et al[34] | 1 | 1 | 1 | 1 | 0 | 1 | 1 | 1 | 7 | 87.5 | medium |
| 4 | Asemahagn[35] | 1 | 1 | 1 | 1 | 1 | 1 | 1 | 1 | 8 | 100 | high |
| 5 | Belachew et al[36] | 0 | 1 | 1 | 1 | 0 | 1 | 1 | 1 | 6 | 75 | medium |
| 6 | Biks et al[37] | 1 | 1 | 1 | 1 | 1 | 1 | 1 | 1 | 8 | 100 | high |
| 7 | Tariku et al[38] | 1 | 1 | 0 | 0 | 0 | 1 | 1 | 1 | 5 | 62.5 | low |
| 8 | Asfaw et al[39] | 1 | 1 | 0 | 0 | 1 | 1 | 1 | 1 | 6 | 75 | medium |
| 9 | Yeshamble Sinshaw et al.[40] | 1 | 1 | 0 | 0 | 0 | 1 | 1 | 1 | 5 | 62.5 | low |
| 10 | Mekuria et al[41] | 1 | 1 | 1 | 1 | 0 | 1 | 1 | 1 | 7 | 87.5 | medium |
| 11 | Arage et al[42] | 1 | 1 | 1 | 1 | 0 | 1 | 1 | 1 | 7 | 87.5 | medium |
| 12 | Gebrie et al[43] | 1 | 1 | 1 | 1 | 1 | 1 | 1 | 1 | 8 | 100 | high |
| 13 | Chekol et al[44] | 1 | 1 | 0 | 0 | 1 | 1 | 1 | 1 | 6 | 75 | medium |
| 14 | Hunegnaw et al.[45] | 1 | 1 | 0 | 0 | 1 | 1 | 1 | 1 | 6 | 75 | medium |
| 15 | Tewabe et al[46] | 0 | 1 | 1 | 1 | 0 | 1 | 1 | 1 | 6 | 75 | medium |
| 16 | Iffa et al[47] | 0 | 1 | 0 | 0 | 0 | 1 | 1 | 1 | 4 | 50 | low |
| 17 | Bayissa Z B. et al[48] | 1 | 1 | 1 | 1 | 1 | 1 | 1 | 1 | 8 | 100 | high |
| 18 | Kitesa et al[49] | 1 | 1 | 1 | 1 | 1 | 1 | 1 | 1 | 8 | 100 | high |
| 19 | Sasie D et al[50] | 0 | 1 | 0 | 0 | 1 | 1 | 1 | 1 | 5 | 62.5 | low |
| 20 | Anjullo B et al[51] | 1 | 1 | 0 | 0 | 1 | 1 | 1 | 1 | 6 | 75 | medium |
| 21 | Muze Edris MD, et al[52] | 1 | 1 | 0 | 0 | 0 | 1 | 1 | 1 | 5 | 62.5 | low |
| 22 | Gedion Asnake Azeze et al.[53] | 1 | 1 | 0 | 0 | 0 | 1 | 1 | 1 | 5 | 62.5 | low |
| 23 | Sorato M[54] | 1 | 1 | 1 | 1 | 1 | 1 | 1 | 1 | 8 | 100 | high |
| 24 | Reddy S et al[55] | 0 | 1 | 1 | 1 | 1 | 1 | 1 | 1 | 7 | 87.5 | medium |
| 25 | Bisrat et al[56] | 1 | 1 | 1 | 1 | 0 | 1 | 1 | 1 | 7 | 87.5 | medium |
| 26 | Sonko A et al[57] | 1 | 1 | 1 | 1 | 1 | 1 | 1 | 1 | 8 | 100 | high |
| 27 | Adugna et al[58] | 1 | 1 | 0 | 0 | 0 | 1 | 1 | 1 | 5 | 62.5 | low |
| 28 | Alemu Earsido.et al[59] | 1 | 1 | 1 | 1 | 0 | 1 | 1 | 1 | 7 | 87.5 | medium |
| 29 | Eskezyiaw Agedew Getahu.et al[60] | 1 | 1 | 1 | 1 | 0 | 1 | 1 | 1 | 7 | 87.5 | medium |
| 30 | Lenja et al[61] | 1 | 1 | 0 | 0 | 0 | 1 | 1 | 1 | 5 | 62.5 | low |
| 31 | Kelaye T[62] | 0 | 1 | 1 | 1 | 1 | 0 | 1 | 1 | 6 | 75 | medium |
| 32 | Tadesse et al[63] | 1 | 1 | 1 | 1 | 1 | 1 | 1 | 1 | 8 | 100 | high |
| 33 | Teka et al[64] | 1 | 1 | 0 | 0 | 1 | 1 | 1 | 1 | 6 | 75 | medium |
| 34 | Shifraw et al[65] | 1 | 1 | 1 | 1 | 0 | 1 | 1 | 1 | 7 | 87.5 | medium |
| 35 | Elyas l[66] | 1 | 1 | 1 | 1 | 1 | 1 | 1 | 1 | 8 | 100 | high |
| 36 | Ahmed et al[67] | 1 | 1 | 1 | 1 | 1 | 1 | 1 | 1 | 8 | 100 | high |
| 37 | Nur et al[68] | 1 | 1 | 1 | 1 | 1 | 1 | 1 | 1 | 8 | 100 | high |
| 38 | Tilksew Ayalew[69] | 1 | 1 | 1 | 1 | 1 | 1 | 1 | 1 | 8 | 100 | high |
| 39 | Alebachew et al[70] | 1 | 1 | 1 | 1 | 1 | 1 | 1 | 1 | 8 | 100 | high |
| 40 | Desalew et al[71] | 0 | 1 | 1 | 1 | 0 | 1 | 1 | 1 | 6 | 75 | medium |
| 41 | Bazie et al[72] | 1 | 1 | 1 | 1 | 1 | 1 | 1 | 1 | 8 | 100 | high |
| 42 | Dibisa et al[73] | 1 | 1 | 1 | 1 | 1 | 1 | 1 | 1 | 8 | 100 | high |
| 43 | Musse Obsiye[74] | 1 | 1 | 1 | 1 | 1 | 1 | 1 | 1 | 8 | 100 | high |
| 44 | Mamo et al[75] | 0 | 1 | 0 | 0 | 1 | 1 | 1 | 1 | 5 | 62.5 | low |
| 45 | Hagos et al[76] | 1 | 1 | 1 | 1 | 1 | 1 | 1 | 1 | 8 | 100 | high |
| We included all primary studies with a quality score of ≥50% in the analysis  Overall appraisal: 45 primary studies  Included: 45 primary studies with a quality score of ≥50% | | | | | | | | | | | |  |
